# Prevalence of blood-borne viral and sexually transmitted infections among homeless people in Berlin

**DOI:** 10.1093/eurpub/ckac131.201

**Published:** 2022-10-25

**Authors:** G Steffen, C Weber, C Cawley, A Leicht, N Sarma, K Jansen, S Kröger, K Kajikhina, R Zimmermann, V Bremer

**Affiliations:** 1 Robert Koch Institute, Berlin, Germany; 2 BeSog Sozialprojekte GgmbH, Berlin, Germany; 3 Checkpoint BLN, Berlin, Germany; 4 Fixpunkt e.V., Berlin, Germany

## Abstract

**Background:**

Risk factors associated with precarious living conditions make people experiencing homelessness (PEH) also highly vulnerable for blood-borne viral and sexually transmitted infections (BBVSTI) and tuberculosis (TB). The number of PEH in Germany is rising, yet little data is available on the infectious burden among this population. A pilot study assessed the prevalence of BBVSTI, TB, behaviours and access to medical services among PEH.

**Methods:**

We recruited PEH from April-June 2021 in five low-threshold medical services in Berlin. Behavioural data was collected via questionnaire-based interviews. Serological/molecular testing from venous blood samples was performed for Hepatitis B (HBV), Hepatitis C (HCV), HIV, syphilis and TB and from urine for Neisseria gonorrhoeae (NG) and Chlamydia trachomatis (CT).

**Results:**

Of 216 participants, 88% (191/216) were male and 73% (158/215) were born abroad. Mean age was 41 years (range 19-68). No health insurance was reported by 57% (123/216) and previous incarceration by 71% (153/214). Of all, 53% (114/216) injected drugs in the last 30 days, and 41% (89/216) reported unprotected sex in the last 12 months. Prevalence of active HBV was 1.9% (4/212), of active HCV 15.9% (34/213), and of HIV 2.8% (6/213). No active TB was diagnosed, while 14.4% (31/216) tested positive for latent TB infection. Active syphilis was found in 1.4% (3/212), NG in 2.0% (4/197), CT in 3.0% (6/197), and serological evidence of HBV vaccination in 26% (56/212). While 44% (96/216) of participants were ever tested for HCV, 71% (36/51) of those with HCV antibodies knew about their infection, 36% (13/36) of them reported previous/current treatment.

**Conclusions:**

Burden of HCV and HIV was high among PEH in Berlin, and risk behaviours were frequently reported. There is a need to improve access to regular health care, accompanied by low-threshold prevention offers in cooperation with drug and homeless services. A nationwide expansion of the study is planned.

**Key messages:**

• High burden of Hepatitis C and HIV among people experiencing homelessness in Berlin, Germany.

• Access of people experiencing homelessness to regular health care needs improvement, accompanied by low-threshold prevention offers in cooperation with drug and homeless services.

